# Potential Root Foraging Strategy of Wheat (*Triticum aestivum* L.) for Potassium Heterogeneity

**DOI:** 10.3389/fpls.2018.01755

**Published:** 2018-11-27

**Authors:** Li Ruan, Xiuli Xin, Jiabao Zhang, Bingzi Zhao, Hao Cheng, Congzhi Zhang, Donghao Ma, Lin Chen

**Affiliations:** ^1^State Key Laboratory of Soil and Sustainable Agriculture, Institute of Soil Science, Chinese Academy of Sciences, Nanjing, China; ^2^National Center for Tea Improvement, Tea Research Institute, Chinese Academy of Agricultural Sciences, Hangzhou, China; ^3^Key Laboratory of Tea Plant Biology and Resources Utilization, Ministry of Agriculture, Hangzhou, China

**Keywords:** K heterogeneity, root foraging strategy, root morphology, root gene expression, K^+^ flux, O_2_ flux

## Abstract

Potassium (K) distribution is horizontally heterogeneous under the conservation agriculture approach of no-till with strip fertilization. The root foraging strategy of wheat for K heterogeneity is poorly understood. In this study, WinRHIZO, microarray, Non-invasive Micro-test Technology (NMT) and a split-root system were performed to investigate root morphology, gene expression profiling and fluxes of K^+^ and O_2_ under K heterogeneity and homogeneity conditions. The split-root system was performed as follows: C. LK (both compartments had low K), C. NK (both compartments had normal K), Sp. LK (one compartment had low K) and Sp. NK (the other compartment had normal K). The ratio of total root length and root tips in Sp. NK was significantly higher than that in C. NK, while no significant differences were found between Sp. LK and C. LK. Differential expression genes in C. LK vs. C. NK had opposite responses in Sp. LK vs. C. LK and similar responses in Sp. NK vs. C. NK. Low-K responsive genes, such as peroxidases, mitochondrion, transcription factor activity, calcium ion binding, glutathione transferase and cellular respiration genes were found to be up-regulated in Sp. NK. However, methyltransferase activity, protein amino acid phosphorylation, potassium ion transport, and protein kinase activity genes were found to be down-regulated in Sp. LK. The up-regulated gene with function in respiration tended to increase K^+^ uptake through improving O_2_ influx on the root surface in Sp. NK, while the down-regulated genes with functions of K^+^ and O_2_ transport tended to reduce K^+^ uptake on the root surface in Sp. LK. To summarize, wheat roots tended to perform active-foraging strategies in Sp. NK and dormant-foraging strategies in Sp. LK through the following patterns: (1) root development in Sp. NK but not in Sp. LK; (2) low-K responsive genes, such as peroxidases, mitochondrion, transcription factor activity, calcium ion binding and respiration, were up-regulated in Sp. NK but not in Sp. LK; and (3) root K^+^ and O_2_ influxes increased in Sp. NK but not in Sp. LK. Our findings may better explain the optimal root foraging strategy for wheat grown with heterogeneous K distribution in the root zone.

## Introduction

Potassium (K), which accounts for 2–10% of the plant’s total dry weight, is one of the most important macronutrients for crop growth (Leigh and Wyn Jones, 1984; Ma et al., 2012; Ruan et al., 2015). K is heterogeneously distributed in soils under minimum tillage (Jackson and Caldwell, 1993; Richard et al., 2000). As a major conservation agriculture approach, no-till has been adopted on 180 million hectares (Mha), or almost 12.5% of the global arable land area (Kassam et al., 2018).In China, no-till, which is usually combined with strip fertilization, is being increasingly advocated for wheat. Approximately 24% of the wheat crop was planted under the agriculture approach of no-till combined with strip fertilization in China by 2009 (Zhao et al., 2017). No-till combined with strip fertilization increases the horizontal spatial variation of K in soils (e.g., a higher K concentration on the fertilized sides and a lower K concentration on the unfertilized sides). This spatial variation occurs due to the wide-row spacing, the fixed crop rows and the fertilizer bands, the high crop residue concentration on the crop rows, the differences in soil properties in relation to their position in the crop-row, and limited K mobility in dry land soils (Ma et al., 2007; Farmaha et al., 2011, 2012; Fernández et al., 2011, 2015; Williams et al., 2017). In rainfed systems, such as those of wheat growing areas in Northern China, the horizontal spatial variation of K is more noteworthy since soil drying conditions generally occur at the end of the growing season when K and water are in greatest demand by wheat (Fernández et al., 2011; Zhao et al., 2017).

Under heterogeneous K distribution, root systems have foraging behaviors for K (Hodge, 2004; Li et al., 2017). In the dry season, K presents relatively low mobility when drying reduces soil water and increases the diffusion path length of K ions to the root surface (Römheld and Kirkby, 2010; Fernández et al., 2011). Root morphology characteristics (especially the root tips, root surface area and root length) are the criteria for evaluating root abilities of penetrating soil and contacting K when K presents with relatively low mobility (Hassan and Arshad, 2010). Root systems can enhance their length and surface area in the soil K-rich patches and restrict them in K-deficient patches (Fernández et al., 2011; Kellermeier et al., 2013). The underlying molecular mechanisms, which are involved in the root morphological changes in response to K heterogeneity, are mainly dependent on the regulation of genes encoding hormones. These hormone genes mainly include auxin, ethylene and jasmonic acid-related genes (Jung et al., 2009; Liu et al., 2013; Giehl et al., 2014; Gupta et al., 2017; Lee et al., 2017). Root development-related genes are also involved in the root morphological changes in response to K heterogeneity (Giehl and von Wirén, 2014). In addition, other physiological changes, such as the release of nutrient mobilizing root exudates or the expression of nutrient transporters, also provide contributions to root adaptions to K heterogeneity (Gruber et al., 2013; Giehl and von Wirén, 2014; Zhao et al., 2016).

Compared with root foraging strategies for K heterogeneity, root foraging strategies for nitrogen (N) and phosphorus (P) heterogeneity are more abundant. In addition to the regulation of root morphology, root exudates, nutrient transporters, hormone genes and root development-related genes (Shen et al., 2005; Paterson et al., 2006; Guan et al., 2014; Sun et al., 2018), other physiological and molecular changes have also been found to be involved in the root foraging strategy for N and P heterogeneity. Cytoskeleton activation, cell wall modification, arbuscular mycorrhizal fungi and so on are involved in root foraging for N and P heterogeneity (Duan et al., 2015; Trevisan et al., 2015). Various genes, such as genes involved in cell expansion and division, transcription factors, kinases, sugar transportation and utilization, NO_3_^-^ absorption and assimilation, cytokinin biosynthesis and so on, have been proven to be involved in root foraging responses to N and P heterogeneity (Liu et al., 2008; Krouk et al., 2010; Ruffel et al., 2011; Alvarez et al., 2012; Robaglia et al., 2012; Tabata et al., 2014; Shabnam and Iqbal, 2016; Zhou et al., 2017; Haling et al., 2018). However, unlike N and P, K does not convert into other organic compounds in plants. Thus, K^+^ can transport between tissues in plants. K heterogeneity results in the roots of the same plant being exposed to different K conditions. K^+^ in the root exposed to a low-K condition may be supplemented by K^+^ absorbed by a root exposed to a high-K condition. Therefore, root foraging strategies in the presence of K heterogeneity may be quite different from that for N or P. In most cases, the transport of K^+^ between tissues involves active transport, which requires a lot of energy (Szczerba et al., 2009). Aerobic respiration, which provides energy, may play a key role in dealing with K heterogeneity. However, aerobic respiration is rarely considered when studying root forging strategies for K heterogeneity.

Therefore, we proposed that plants could realize the maximum utilization of energy and the optimum state of the root systems through root foraging behaviors under K heterogeneity (Mcnickle et al., 2009). In order to investigate the optimal root foraging strategy of wheat for K heterogeneity, we selected a K efficient wheat genotype “Tongzhou916” as the research object, which has a high K efficiency coefficient, great root development, strong K uptake ability and up-regulation of low-K responsive genes (Ruan et al., 2015). Because using localized fertilization in row crops is a similar phenomenon to the split-root system, we used the split-root system to mimic a heterogeneous soil environment. In this study, root parameters, gene expression profiling and root fluxes of net K^+^ and O_2_ were analyzed by WinRHIZO, Affymetrix GeneChip and NMT, respectively. The results in this study will give us a basis to better understand the optimal root foraging strategy for K heterogeneity in the root zone of wheat.

## Materials and Methods

### Plant Materials and Split-Root System

According to a previous study, Tongzhou916 has the highest K efficiency coefficient among 50 representative wheat varieties. In addition, its great root development, strong K uptake ability and the up-regulation of low-K responsive genes make a great contribution to the low-K tolerance of Tongzhou916 (Ruan et al., 2015). Therefore, “Tongzhou916” was selected as a low-K^+^ tolerant wheat genotype and used as the test object in this research. The hydroponics test of the wheat was carried out in an artificial climate box. The specific environmental parameters are shown below: (1) the day and night time were 16 and 8 h, respectively; (2) the day and night temperature were 25 and 18°C, respectively; (3) the illumination intensity was 30000 lux in the day; (4) the atmospheric humidity was 70%.

In order to simulate heterogeneous soil K availability, we used a split-root system. Two separated physical spaces, in which different K supplies could be provided, were created in this split-root system. During the split-root experiment, we selected wheat seedlings with the same growth of roots and shoots. Then, the wheat roots were planted on two separated physical spaces uniformly. Each separated physical space contained the same root tip number and root length at the beginning of the experiment. Three different K environments were provided as follows (Ruffel et al., 2011): (1) a homogeneous K-deprived condition (C. LK: both compartments had low K), (2) a homogeneous K-replete condition (C. NK: both compartments had a normal K), and (3) a heterogeneous split condition (Sp. LK/Sp. NK: one compartment had low K, and the other had normal K). The nutrition solution was (mmol L^-1^): NH_4_NO_3_ 1.0, Ca(NO_3_)_2_⋅4H_2_O 1.0, NaH_2_PO_4_ 0.25, MgSO_4_⋅H_2_O 1.0, CaCl_2_ 1.5, Fe-EDTA 0.1, ZnSO_4_⋅7H_2_O 1.0 × 10^-3^, (NH_4_)_6_ Mo_7_O_24_⋅4H_2_O 5.0 × 10^-5^, MnSO_4_⋅H_2_O 1.0 × 10^-3^, CuSO_4_⋅5H_2_O 5.0 × 10^-4^, and H_3_BO_4_ 1.0 × 10^-3^. The normal K treatment contained 1.0 mmol L^-1^ K_2_SO_4_, while the low K treatment contained 0.005 mmol L^-1^ K_2_SO_4_. All of the treatments had a pH of 6.5. The nutrient solution was changed once a day. The nutrient solution was supplied 12 h O_2_/d by pumps.

In the first experiment, to analyze the shoot dry weight, K biological utilization and root development, seedlings were transferred to the split-root system for 4 weeks. Then, the seedlings were harvested, and we investigated the phenotypes, shoot dry weights, K contents and root parameters. In the second experiment, for the investigation of the molecular basis of the wheat root responses to heterogeneous vs. homogeneous K environments, 3-week-old seedlings, which were first planted in normal K solutions, were transplanted to the above split-root system for 5 days. Then, the wheat roots on each side were collected. The harvested roots were put into liquid N quickly to prepare them for subsequent RNA isolation. The split-root system contained four treatments as follows: (1) a homogeneous K-deprived condition (C. LK: both compartments had 0.005 mmol L^-1^ K_2_SO_4_), (2) a homogeneous K-replete condition (C. NK: both compartments had 1.0 mmol L^-1^ K_2_SO_4_), (3) a heterogeneous split condition (Sp. LK/Sp. NK: one compartment had 0.005 mmol L^-1^ K_2_SO_4_, and the other had 1.0 mmol L^-1^ K_2_SO_4_), and (4) another heterogeneous split condition (Sp_0_. MK/Sp_0_. NK: one compartment had 0.5 mmol L^-1^ K_2_SO_4_, and the other had 1.0 mmol L^-1^ K_2_SO_4_).

### Root Parameters, Biomass and K^+^ Content Measurement

An optical scanner (Epson, Japan) was used to scan the whole roots. WinRHIZO was performed to analyze the root parameters. In order to eliminate the influence of light and temperature in the day and night, we took samples at two time points during the day and night. We harvested the roots and shoots of the test plants separately. Then, the harvested roots and shoots were dried in an oven at a temperature of 80°C for 48 h. After drying, the dry weight of the shoots and roots was obtained. The plant K^+^ content was measured after digesting the material with a mixture of H_2_O_2_ and H_2_SO_4_ (Mills and Jones, 1996) followed by flame photometer to measure the K^+^ content of the digested solution. For the plant biomass and K^+^ concentration measurement experiments, three biological replications were performed (each replicate included 20 individual plants). The formula of the K biological utilization is shown below:

K biological utilization = dry weight of shoot/K concentration of shoot (1).

### Microarray Analysis

With the help of the Shanghai Biotechnology Corporation, the microarray analysis was performed successfully. Three biological replicates (each replicate included 20 individual plants) were carried out in this experiment. According to the instructions of the manufacturer, we extracted the total RNA by using TRIzol reagent (Cat#15596-018, Life Technologies, Carlsbad, CA, United States). With the help of Agilent Bioanalyzer 2100 (Agilent Technologies, Santa Clara, CA, United States), we checked the integrity of the RNA by determining its RIN number. We purified the total RNA by using an RNeasy micro kit (Cat#74004, QIAGEN, GmBH, Germany) and RNase-Free DNase Set (Cat#79254, QIAGEN, GmBH, Germany) (Ruan et al., 2015).

According to the instructions of the manufacturer, we amplified, labeled and purified the total RNA through the use of the GeneChip 3^′^IVT Express Kit (Cat#901229, Affymetrix, Santa Clara, CA, United States) in order to obtain biotin labeled cRNA (Wu et al., 2013). According to the instructions of the manufacturer, we performed array hybridization and washing by carrying out GeneChip^®^ Hybridization, Wash and Stain Kit (Cat#900720, Affymetrix, Santa Clara, CA, United States) in a Hybridization Oven 645 (Cat#00-0331-220V, Affymetrix, Santa Clara, CA, United States) and Fluidics Station 450 (Cat#00-0079, Affymetrix, Santa Clara, CA, United States). We scanned the slides by using a GeneChip^®^ Scanner 3000 (Cat#00-00212, Affymetrix, Santa Clara, CA, United States) and Command Console Software 3.1 (Affymetrix, Santa Clara, CA, United States) with default settings (Zeng et al., 2014; Zhu et al., 2016).

### Determination of the Flux Rates of K^+^ and O_2_ on the Wheat Root Surfaces

In order to determine the K^+^ and O_2_ net flux rates on the wheat root surfaces, wheat roots were cut off from the whole root system of each plant. Before detection, we transferred the wheat roots to a Petri dish that contained 10 mL of testing solution. After 10 min of incubation to allow them to adapt and reduce any instability induced by environmental changes, the wheat roots were used for measurements. During the detection stage, we transferred the balanced roots to a new Petri dish that contained a fresh testing solution. K^+^ flux and O_2_ influx were determined by utilizing NMT (Non-invasive Micro-test System, BIO-001A; Younger USA LLC., Amherst, MA, United States). Six repeats were set for each test treatment. The net K^+^ flux rates along the root axis (from 0 to 250 mm) were measured to determine the maximal net K^+^ influx areas along the root axis (Supplementary Figure S1). In the areas of 7 cm to 10 cm from the root apex, we found that the K^+^ net flux rate reached the peak value. Therefore, the areas between 7 and 10 cm from the root apex were selected as the measurement site. The determining time for each tested root was 10 min. The measurement details of ion flux, such as the NMT system and the related applications, have been reported in previous research (Zheng et al., 2012; Ruan et al., 2016; Zhang et al., 2016).

### Quantitative Real-Time PCR (qRT-PCR) Analysis

To verify the reliability of our microarray, Quantitative Real-time PCR (qRT-PCR) was performed. The testing roots for qRT-PCR were the part of the roots used for the microarray experiment. The total RNA extractions of root samples were performed by using the method for the TRIZOL reagent (Cat#15596-018, Life technologies, Carlsbad, CA, United States). The endogenous control used in this experiment was the actin gene of *Triticum aestivum* L. According to the instructions of the manufacturer, we synthesized first-strand cDNA by using the extracted total RNA with a cDNA synthesis Kit (Promega, United States). Three biological replicates (each replicate included 20 individual plants) were carried out in this experiment. A 20 μL reaction system, which contained 10, 6, 2, 1, and 1 μL of 2X-RT mix, nuclease-free water, template (0.2 μM), forward primer and reverse primer, was used to carry out the experiment of qRT-PCR. In the experiment of the qRT-PCR, the software of Primer 5 and DNAMAN were used to design the PCR primers (Supplementary Table S1). At least three technical duplications were performed for each PCR experiment. The relative expression of the chosen genes was calculated through the relative quantitative method of ΔΔ*C*T (Revel et al., 2002; Ruan et al., 2015).

### Statistical Analysis

The data of the microarray were normalized through the utilization of the software of MAS 5.0 algorithm, Gene Spring Software 11.0 (Agilent technologies, Santa Clara, CA, United States). We only selected the genes that showed significant differences (*P* < 0.05 and fold-change > 1.5) as further research objects. The data analysis was carried out with the help of SBC Analysis System^1^. The functional annotation was performed with the help of AgriGO online service^2^. For figure drawing, OriginPro 8.1 (Origin Inc., Chicago, IL, United States) was used. The significance of the group differences was calculated by using ANOVA and *t*-tests. Microsoft Excel (Microsoft Corporation, United States) and SPSS 18 (SPSS Inc., Chicago, IL, United States) were performed for the data statistical analysis.

## Results

### Wheat Growth State Under the Split-Root System

**FIGURE 1:**
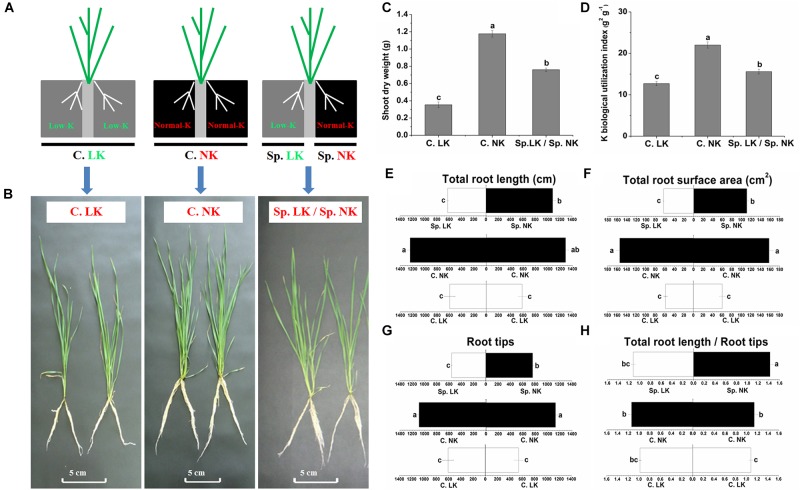
Phenotype, shoot dry weight, K biological utilization and root development of wheat under a split-root system. **(A)** Diagram showing the split-root system used to detect potassium (K) foraging behavior of wheat root. Two separated physical spaces, in which different K supplies could be applied, were created in this split-root system. Three different K environments were used as follows: (1) a homogeneous K-deprived condition (C. LK: both compartments had low K), (2) a homogeneous K-replete condition (C. NK: both compartments had normal K), and (3) a heterogeneous split condition (Sp. LK/Sp. NK: one compartment had low K, and the other had normal K). Phenotype **(B)**, shoot dry weight **(C)**, K biological utilization **(D)** of wheat under a split-root system. Total root length **(E)**, total root surface area **(F)**, root tips **(G)**, ratio of total root length and root tips **(H)**. Data are shown as means ± SE (*n* = 3). Different letters showed significant differences between pairs of treatments (i.e., C. LK vs. C. NK, Sp. NK vs. C. NK, Sp. LK vs. C. LK, Sp. LK vs. Sp. NK) at the level of *P* < 0.05.

In order to simulate heterogeneous soil K distribution, we used a split-root system in the current research. Two separated physical spaces, in which different K supplies could be provided, were created in this split-root system. To study the potassium (K) foraging behavior of wheat roots, we designed three treatments (Figure 1A). The phenotypic differences were significant between all of the treatments (Figure 1B). Significant differences in shoot dry weight and K biological utilization were found among all of the treatments (Figures 1C,D). Shoot dry weight and K biological utilization were ranked as follows: C. NK > Sp. LK/Sp. NK > C. LK. Shoot dry weight and K biological utilization in the treatment of Sp. LK/Sp. NK were 64.6 and 70.8% of values for C. NK, respectively. In addition, there were significant differences in root development among any of the treatments (Figures 1E–H). The root system grew uniformly in homogeneous K environments (C. LK and C. NK) and non-uniformly in a heterogeneous split environment (Sp. LK/Sp. NK). The total root length, total root surface area and root tips in C. LK were 46.6, 38.0, and 51.8% of those in C. NK (Figures 1E–G). The total root length, total root surface area and root tips in Sp. NK were 85.9, 71.1, and 68.8% of those in C. NK (Figures 1E–G). The ratio of total root length and root tips in Sp. NK was significantly higher than that in C. NK (Figure 1H). No significant differences were found in root development between Sp. LK and C. LK (Figures 1E–H). The total root length, total root surface area and root tips in Sp. NK were more than half of those in C. NK (Figures 1E–G). The ratio of total root length and root tips in Sp. NK was significantly higher than that in C. NK (Figure 1H). In addition, we compared two types of K heterogeneity as follows: (a) Sp_0_. MK/Sp_0_. NK: one compartment had 0.5 mmol L^-1^ K_2_SO_4_, and the other had 1.0 mmol L^-1^ K_2_SO_4_; (b) Sp. LK/Sp. NK: one compartment had 0.005 mmol L^-1^ K_2_SO_4_, and the other had 1.0 mmol L^-1^ K_2_SO_4_. The results showed that the total root length, total root surface area and root tips in Sp. NK were significantly higher than those in Sp_0_. NK (Supplementary Table S2). The ratio of total root length and root tips in Sp. NK was also significantly higher than that in Sp_0_. NK (Supplementary Table S2).

### Differentially Expressed Genes (DEGs) in Wheat Root Responses to Heterogeneous vs. Homogeneous K Environments

In this study, more than 61200 probe signals were detected from the Affymetrix GeneChip. The raw data sets (CEL) and the normalized expression data sets have been deposited in the Gene Expression Omnibus (GSE115111) at the National Center for Biotechnology Information^3^. For further analysis, the genes with significant differences (*P* < 0.05 and fold-change > 1.5) were chosen to include as many candidate genes as possible.

**FIGURE 2:**
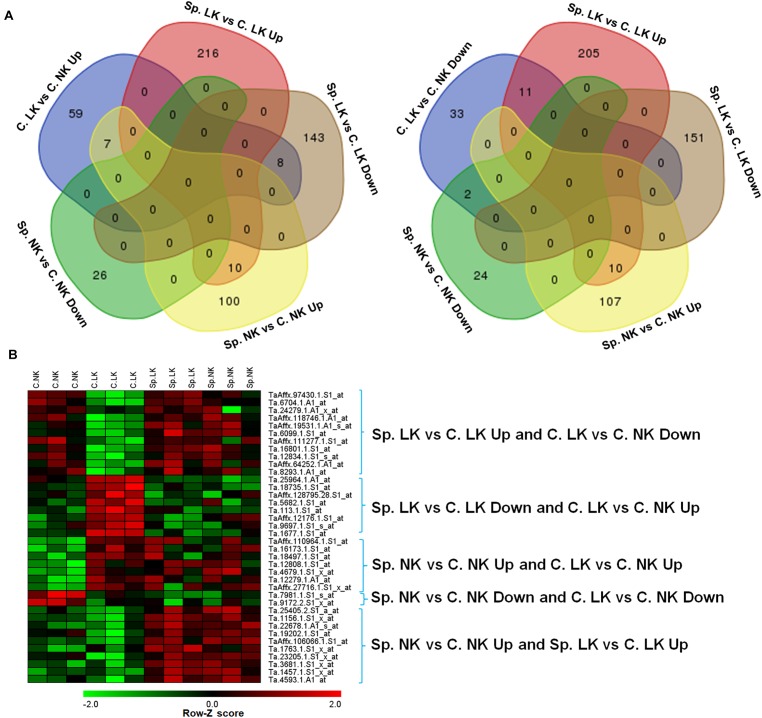
**(A)** Venn diagrams of DEGs in wheat root responses to heterogeneous vs. homogeneous K environments. **(B)** Heat map of DEGs in wheat root responses to heterogeneous vs. homogeneous K environments. This heat map showed the interaction of DEGs between K heterogeneous and homogeneous environments in ANOVA. All microarray experiments were carried out in three biological replications.

There were 59 up-regulated genes in C. LK vs. C. NK. Among these genes, there were 8, 7, 0, and 0 shared genes in Sp. LK vs. C. LK down-regulated genes, Sp. NK vs. C. NK up-regulated genes, Sp. LK vs. C. LK up-regulated genes, and Sp. NK vs. C. NK down-regulated genes, respectively (Figure 2A). There were 33 down-regulated genes in C. LK vs. C. NK. Among these genes, there were 11, 2, 0, and 0 shared genes in Sp. LK vs. C. LK up-regulated genes, Sp. NK vs. C. NK down-regulated genes, Sp. LK vs. C. LK down-regulated genes, and Sp. NK vs. C. NK up-regulated genes, respectively (Figure 2A). There were 10 up-regulated genes shared in Sp. LK vs. C. LK and Sp. NK vs. C. NK (Figure 2A). The heat map of the above shared genes is shown in Figure 2B. Differential expression of genes in C. LK vs. C. NK had opposite responses in Sp. LK vs. C. LK and similar responses in Sp. NK vs. C. NK (Figure 2B).

### Functional Annotations of Differentially Expressed Genes in Wheat Root Responses to Heterogeneous vs. Homogeneous K Environments

**FIGURE 3:**
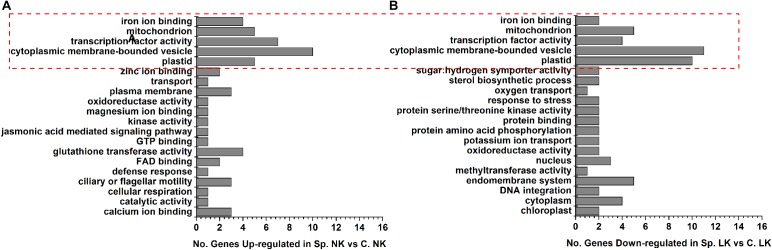
Gene Ontology (GO) classifications and amount of DEGs in wheat root response to heterogeneous vs. homogeneous K environments. **(A)** Functional category distributions of main up-regulated genes in Sp. NK vs. C. NK. **(B)** Functional category distributions of main down-regulated genes in Sp. LK vs. C. LK. All microarray experiments were performed in three biological replicates.

To study the functions of putative key genes of wheat in response to heterogeneous vs. homogeneous K environments (Supplementary Table S3), Gene Ontology (GO) analysis was performed to analyze the functional differences between differentially expressed genes in Sp. NK vs. C. NK and Sp. LK vs. C. LK (Figure 3). There were five shared functions of up-regulated genes in Sp. NK vs. C. NK and down-regulated genes in Sp. LK vs. C. LK, including iron ion binding, mitochondria, transcription factor activity, cytoplasmic membrane-bounded vesicle and plastids (Figure 3). In addition, functional categories of up-regulated genes unique in Sp. NK vs. C. NK included calcium ion binding, glutathione transferase, cellular respiration, jasmonic acid mediated signaling pathway and so on (Figure 3A). Functional categories of down-regulated genes unique in Sp. LK vs. C. LK included methyltransferase activity, protein amino acid phosphorylation, potassium ion transport, protein serine/threonine kinase activity and so on (Figure 3B).

### Unique Differentially Expressed Genes and Net Flux Rates of K^+^ and O_2_ in Heterogeneous vs. Homogeneous K Environments

**FIGURE 4:**
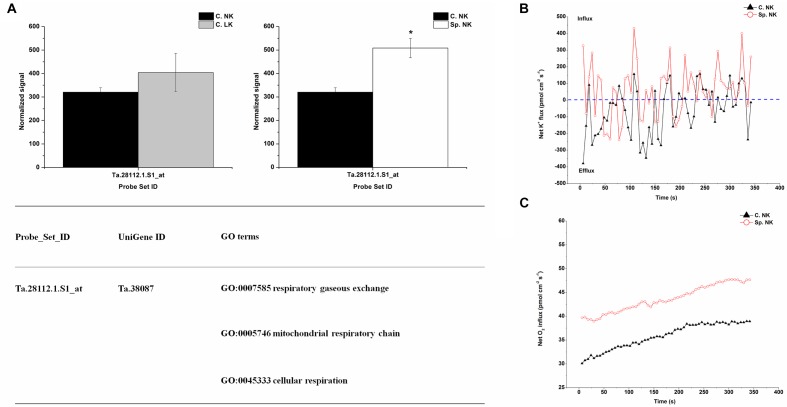
Unique differentially expressed gene and net flux rates of K^+^ and O_2_ in heterogeneous vs. homogeneous normal-K environments. **(A)** Expression pattern and GO classifications of unique differentially expressed genes in heterogeneous (Sp. NK) vs. homogeneous (C. NK) normal-K environments. The means ± SD (*n* = 3) are shown. ^∗^ indicates differences between means at *P* < 0.05. All microarray experiments were performed in three biological replicates. **(B)** The rates of net K^+^ fluxes in Sp. NK and C. NK. **(C)** The rates of net O_2_ fluxes in Sp. NK and C. NK. Six biological replicates were established in the net flux experiments.

In order to investigate the key genes involved in K uptake, unique up-regulated genes in Sp. NK vs. C. NK was shown in Figure 4A. The unique genes had the following functions: respiratory gaseous exchange, mitochondrial respiratory chain and cellular respiration (Figure 4A). The up-regulated gene in Sp. NK vs. C. NK was up-regulated in C. LK vs. C. NK at the same time. In addition, the fold change in Sp. NK vs. C. NK was higher than that in C. LK vs. C. NK (Figure 4A). To compare the flux rate of K^+^ and O_2_ on the root surface in Sp. NK and C. NK, NMT was performed in this research. The net flux rate of K^+^ and O_2_ in Sp. NK and C. NK were shown in Figures 4B,C. For flux rate of K^+^, the curves presented vibration trends for both Sp. NK and C. NK (Figure 4B). The K^+^ flux presented alternate states of influx and efflux for both Sp. NK and C. NK. However, the overall influx rate of K^+^ in Sp. NK was higher than that in C. NK. For flux rate of O_2_, the curves presented gradual upward trends for both Sp. NK and C. NK (Figure 4C). For both Sp. NK and C. NK, the root surfaces presented absorption state. The influx rate of O_2_ in Sp. NK was higher than that in C. NK.

**FIGURE 5:**
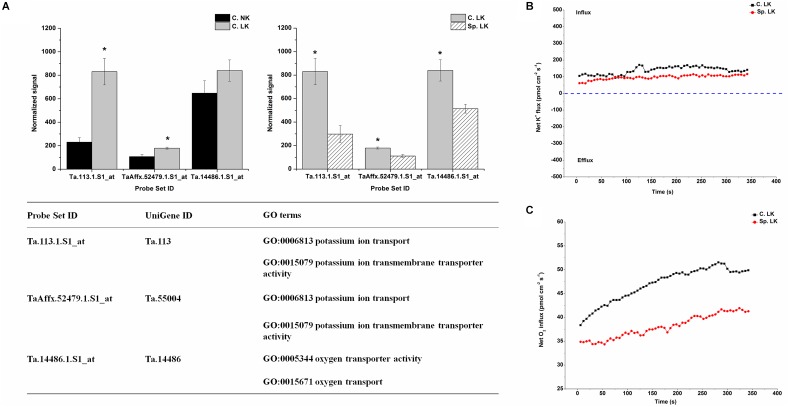
Unique differentially expressed genes and net flux rates of K^+^ and O_2_ in heterogeneous vs. homogeneous low-K environments. **(A)** Expression pattern and GO classifications of unique differentially expressed gene in heterogeneous (Sp. LK) vs. homogeneous (C. LK) low-K environments. Data are means ± SD (*n* = 3). ^∗^ indicates differences between means at *P* < 0.05. All microarray experiments were performed in three biological replicates. **(B)** The rates of net K^+^ fluxes in Sp. LK and C. LK. **(C)** The rates of net O_2_ fluxes in Sp. LK and C. LK. Six biological replicates were established in the net flux experiments.

To investigate the key genes involved in K uptake, unique down-regulated genes in Sp. LK vs. C. LK were shown in Figure 5A. The unique genes had the following functions: potassium ion transport, potassium ion transmembrane transporter activity, oxygen transporter activity and oxygen transport (Figure 5A). In contrast, the down-regulated genes in Sp. LK vs. C. LK were up-regulated in C. LK vs. C. NK (Figure 5A). In order to compare the flux rate of K^+^ and O_2_ on the root surface in Sp. LK and C. LK, NMT was applied in this study. The net flux rate of K^+^ and O_2_ in Sp. LK and C. LK were shown in Figures 5B,C. For the flux rate of K^+^, the curves presented relatively steady absorption states for both Sp. LK and C. LK (Figure 5B). However, the overall influx rate of K^+^ in C. LK was higher than that in Sp. LK. For the flux rate of O_2_, the curves presented gradual upward trends for both Sp. LK and C. LK (Figure 5C). For both Sp. LK and C. LK, the root surfaces presented the absorption state. The influx rate of O_2_ in C. LK was higher than that in Sp. LK.

### Quantitative Real-Time PCR (qRT-PCR) Analysis

**FIGURE 6:**
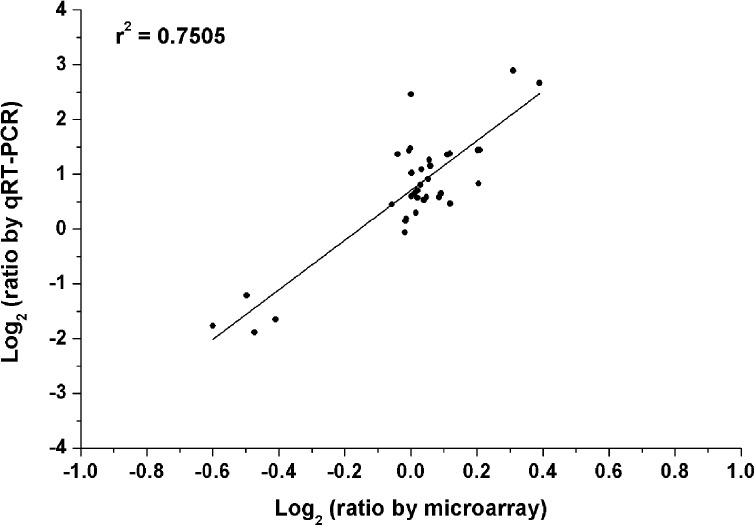
Validation of microarray data by the method of quantitative real-time PCR (qRT-PCR). All microarray and qRT-PCR experiments were carried out in three biological replications. Data are the log_2_ ratio (heterogeneous K/homogeneous K) for the selected genes. The correlation coefficient (*r*^2^) is shown in the diagram.

To verify the reliability of our microarray results, quantitative real-time PCR (qRT-PCR) was carried out. In the test of qRT-PCR, several genes were selected randomly from the above four treatments. The specific-primers of these selected genes were designed as shown in Supplementary Table S1. The data of qRT-PCR showed that gene expression trends were significantly similar (*r*^2^ = 0.75) to the results of the microarray data (Figure 6). This indicated that the results of the microarray results were credible in this study.

## Discussion

As shown above, coordinated physiological and molecular responses suggested that a variety of processes took part in the wheat root responses to heterogeneous vs. homogeneous K environments. Thus, we will focus on the three questions below: (1) What kind of root foraging strategy was optimal for K heterogeneity in wheat? (2) What were the major physiological processes taking part in the adaption to K heterogeneity of wheat? (3) What key genes were involved in regulating the above physiological processes?

The active-foraging strategy in Sp. NK and dormant-foraging strategy in Sp. LK was the optimal root foraging strategy for K heterogeneity in wheat. Such a root foraging strategy made the treatment of Sp. LK/Sp. NK produce more than half of the shoot dry weight and K biological utilization of C. NK, indicating that such a root foraging strategy could effectively improve K uptake/assimilation ability in wheat. This foraging strategy included the modulation of root morphology, the expression of low-K responsive genes, and the variations of root K^+^ and O_2_ flux rates between Sp. NK and Sp. LK.

Root systems have the capacity to detect their external environment and increase their absorption ability through root morphology plasticity (Schachtman and Shin, 2007). The root morphology plasticity, as shown in the increased root length in K-rich patches and the decreased root length in K-deficient patches, has been found in various plant species (Hassan and Arshad, 2010; Fernández et al., 2011; Kellermeier et al., 2013). The higher root length allows roots to penetrate soil and uptake more K^+^ (Hassan and Arshad, 2010). In this study, wheat roots performed the root morphology modulation of increased root length in Sp. NK and decreased root length in Sp. LK. Moreover, the ratio of total root length and root tips (i.e., total root length/root tips) in Sp. NK were significantly higher than that in C. NK, indicating that the length of each root in Sp. NK was significantly higher than that in C. NK. This suggests that roots adopt an “active-foraging strategy” characterized by making each root longer rather than increasing the root number in Sp. NK. No significant differences were found in the root development between Sp. LK and C. LK, indicating the K supply on the side of Sp. NK had little impact on the root development in Sp. LK. This suggested that the roots adopted a “dormant foraging strategy” through inhibiting root elongation in Sp. LK. In addition, both Sp. NK and Sp_0_. NK had 1.0 mmol L^-1^ K_2_SO_4_ (a normal K supply). However, the K supply on the other sides of Sp. NK and Sp_0_. NK were different (i.e., the K supply on the other side of Sp. NK was a low K; the K supply on the other side of Sp_0_. NK was a moderate K). Thus, the larger root system in Sp. NK was promoted by the side of Sp. LK, not the normal K supply itself. Therefore, a low-K supply was more likely to stimulate root growth on the other side with a normal-K supply.

In this study, low-K responsive genes (e.g., differentially expressed genes in C. LK vs. C. NK) were up-regulated in Sp. NK and down-regulated in Sp. LK. This regulation also supported the above root foraging strategy for K heterogeneity. Since low-K responsive genes made great contributions to the strong ability of K deficiency tolerance in plants (Ma et al., 2012; Ruan et al., 2015; Li et al., 2017), low-K responsive genes might improve the wheat low-K tolerance under K heterogeneity by improving the low-K tolerance of wheat roots on the normal-K supply side (Sp. NK), rather than by improving the low-K tolerance of wheat roots on the low-K supply side (Sp. LK). The low-K responsive genes screened in this study included genes previously reported, such as peroxidase, transcription factors, calcium ion binding, glutathione transferase, cellular respiration, jasmonic acid, methyltransferase, protein amino acid phosphorylation, and protein amino acid phosphorylation related genes. These genes play a key role in various processes of low-K responses, such as the production and elimination of reactive oxygen species (ROS), regulation of the high-affinity K^+^ absorption transporters, the promotion of root development, modifications of K transporters or channels, respiration and so on (Armengaud et al., 2004; Mittler et al., 2004; Shin and Schachtman, 2004; Shin et al., 2005; Hong-Hermesdorfa et al., 2006; Lee et al., 2007; Ho and Tasy, 2010; Kim et al., 2010, 2012; Held et al., 2011; Ma et al., 2012; Hong et al., 2013; Wang and Wu, 2013; Hafsi et al., 2014). Moreover, some new low-K responsive genes, such as genes encoding mitochondrial and oxygen transport genes were also screened in this study.

Mitochondria, as the main site for aerobic respiration, are structures that generate most of the ATP in cells (Stoimenova et al., 2007). Therefore, the up-regulations of genes encoding mitochondria could provide energy for K^+^ transmembrane transportation. In the present study, genes encoding mitochondria were up-regulated in Sp. NK vs. C. NK and down-regulated in Sp. LK vs. C. LK, indicating that mitochondria might tend to provide more driving forces for K transmembrane transportation in the treatment of Sp. NK. In addition, the root K^+^ uptake was markedly reduced under conditions of oxygen deprivation, while the K^+^ uptake was improved when the oxygen supply was increased (Mugnai et al., 2011; Pottosin, 2014). In this study, the down-regulation of genes encoding oxygen transport reduced the O_2_ uptake ability in Sp. LK, suggesting that the energy produced by the oxygen transport-mediated aerobic respiration decreased in Sp. LK. Less energy led to a lower K^+^ influx rate in Sp. LK than in C. LK. Above all, the expression patterns of the above genes supported the active-foraging strategy in Sp. NK and a dormant-foraging strategy in Sp. LK.

The major physiological processes taking part in the adaption to K heterogeneity of wheat were the variations of root K^+^ and O_2_ flux rates between Sp. NK and Sp. LK. The root K^+^ uptake was markedly reduced under conditions of oxygen deprivation, while the K^+^ uptake was improved when the oxygen supply was increased (Mugnai et al., 2011; Pottosin, 2014). Therefore, O_2_ played an important role in K^+^ absorption. In the present study, both K^+^ and O_2_ influx rates in Sp. NK were higher than those in C. NK, while both K^+^ and O_2_ influx rates in Sp. LK were lower than those in C. LK. Root aerobic respiration could improve K transport ability by supplying more energy for plants under low-K stress (Singh and Blanke, 2000; Hafsi et al., 2014). The higher O_2_ influx rates provided more energy for K transport in Sp. NK, while the lower O_2_ influx rates led to a weaker K transport ability in Sp. LK. Therefore, the variations in root K^+^ and O_2_ flux rates between Sp. NK and Sp. LK supported the active-foraging strategy in Sp. NK and dormant-foraging strategy in Sp. LK. These physiological processes allow wheat the maximum utilization of energy, which could improve the adaption ability to the K heterogeneity of wheat.

The key genes involved in regulating the above physiological processes include genes with functions of respiratory gaseous exchange, mitochondrial respiratory chains and cellular respiration (Ta.28112.1.S1_at), potassium ion transport (Ta.113.1.S1_at, TaAffx.52479.1.S1_at), and oxygen transport (Ta.14486.1.S1_at). The up-regulation of the genes, which had the functions of respiratory gaseous exchange, mitochondrial respiratory chain and cellular respiration, led to increased O_2_ influx in Sp. NK. The increased O_2_ influx improved root aerobic respiration, which could supply more energy for K transport in Sp. NK (Singh and Blanke, 2000; Hafsi et al., 2014). Indeed, the overall influx rate of K^+^ in Sp. NK was higher than that in C. NK. On the other hand, the down-regulation of genes that have the functions of potassium ion transport and potassium ion transmembrane transporter activity, led to decreased K^+^ influx in Sp. LK.

Various researchers have shown that potassium ion transporters, such as *OsHAK1*, *HvHAK1*, *TaHKT1*, and *AtHAK5*, make great contributions to K^+^ uptake when the external K supply is low (Bañuelos et al., 2002; Garciadeblás et al., 2003; Gierth et al., 2005; Fulgenzi et al., 2008; Ruan et al., 2015). However, the potassium ion transport genes are down-regulated in Sp. LK, which is different from the previous studies performed under homogeneous low-K conditions. The main reason for this difference is that the root system in Sp. LK receives the control of system signals rather than local signals under the K heterogeneity condition. The system signals result in the root system performing the dormant-foraging strategy in Sp. LK. Likewise, the oxygen transport gene was down-regulated in Sp. LK, which could result in the O_2_ influx rate decreasing in Sp. LK. A lower O_2_ influx could make the energy supply for K transport decrease, which would lead to a lower K^+^ influx in Sp. LK. Overall, the key genes involved in regulating the above physiological processes could result in the active-foraging strategy in Sp. NK and dormant-foraging strategy in Sp. LK.

## Conclusion

The root foraging strategy of wheat for K heterogeneity has been reported. Based on the root morphology, root gene expression profiling and root fluxes of net K^+^ and O_2_, it was hypothesized that wheat roots tended to perform active-foraging strategies in Sp. NK and dormant-foraging strategies in Sp. LK through the following patterns: (1) roots elongated in Sp. NK rather than in Sp. LK; (2) low-K responsive genes, such as peroxidases, mitochondria, transcription factor activity, calcium ion binding and respiration, were up-regulated in Sp. NK rather than in Sp. LK; and (3) root K^+^ and O_2_ influxes increased in Sp. NK rather than in Sp. LK. Our findings may help in better understanding of the optimal root foraging strategy of wheat for K heterogeneity. The physiological traits (e.g., root morphology, root flux rates of K^+^ and O_2_) can be used as germplasm screening indicators for high yield wheat under the conservation tillage method of no-till with strip fertilization. In addition, the four key genes, with the functions of root respiration, potassium ion transport and oxygen transport, can be used as a reference for molecular marker assisted breeding for high yield wheat under the conservation agriculture approach of no-till with strip fertilization.

## Author Contributions

JZ and LR designed the experiments. LR and XX conducted the measurements, data analysis, and wrote the manuscript. BZ, HC, CZ, and DM assisted with the data analysis. LC assisted with the experiments. All authors reviewed the manuscript before the submission.

## Conflict of Interest Statement

The authors declare that the research was conducted in the absence of any commercial or financial relationships that could be construed as a potential conflict of interest.

## Footnotes

1 http://sas.shbio.com/portal/root/molnet_shbh/index.jsp

2 http://bioinfo.cau.edu.cn/agriGO

3 http://www.ncbi.nlm.nih.gov/geo/

## Supplementary Material

Figure S1 Net K^+^ fluxes along root tips of wheat.

TABLE S1 Primers used in qRT-PCR experiments.

TABLE S2 Root morphological characteristics under potassium (K) heterogeneity.

TABLE S3 Potential key genes involved in the regulation of wheat root responses to K heterogeneity.

## Abbreviations

C. LK: a homogeneous K-deprived environment with both compartments had low K
C. NK: a homogeneous K-replete environment with both compartments had normal K
NMT: non-invasive Micro-test Technology
Sp. LK: a heterogeneous split environment with the compartment had low K
Sp. NK: a heterogeneous split environment with the compartment had normal K

## References

[B1] Alvarez J. M. Vidal E. A. Gutiérrez R. A. (2012). Integration of local and systemic signaling pathways for plant N responses. *Curr. Opin. Plant Biol.* 15 185–191. 10.1016/j.pbi.2012.03.009 22480431

[B2] Armengaud P. Breitling R. Amtmann A. (2004). The potassium-dependent transcriptome of Arabidopsis reveals a prominent role of jasmonic acid in nutrient signaling. *Plant Physiol.* 136 2556–2576. 10.1104/pp.104.046482 15347784 PMC523322

[B3] Bañuelos M. A. Garciadeblas B. Cubero B. Rodríguez-Navarro A. (2002). Inventory and functional characterization of the HAK potassium transporters of rice. *Plant Physiol.* 130 784–795. 10.1104/pp.007781 12376644 PMC166606

[B4] Duan J. Tian H. Drijber R. A. Gao Y. (2015). Systemic and local regulation of phosphate and nitrogen transporter genes by arbuscular mycorrhizal fungi in roots of winter wheat (*Triticum aestivum* L.). *Plant Physiol. Biochem.* 96 199–208. 10.1016/j.plaphy.2015.08.006 26298806

[B5] Farmaha B. S. Fernández F. G. Nafziger E. D. (2011). No-till and strip-till soybean production with surface and subsurface phosphorus and potassium fertilization. *Agron. J.* 103 1862–1869. 10.2134/agronj2011.0149

[B6] Farmaha B. S. Fernández F. G. Nafziger E. D. (2012). Distribution of soybean roots, soil water, phosphorus and potassium concentrations with broadcast and subsurface-band fertilization. *Soil Sci. Soc. Am. J.* 76 1079–1089. 10.2136/sssaj2011.0202

[B7] Fernández F. G. Brouder S. M. Volenec J. J. Beyrouty C. A. Hoyum R. (2011). Soybean shoot and root response to localized water and potassium in a split-pot study. *Plant Soil* 344 197–212. 10.1007/s11104-011-0740-z

[B8] Fernández F. G. Sorensen B. A. Villamil M. B. (2015). A comparison of soil properties after five years of no-till and strip-till. *Agron. J.* 107 1339–1346. 10.2134/agronj14.0549

[B9] Fulgenzi F. R. Peralta M. L. Mangano S. Danna C. H. Vallejo A. J. Puigdomenech P. (2008). The ionic environment controls the contribution of the barley HvHAK1 transporter to potassium acquisition. *Plant Physiol.* 147 252–262. 10.1104/pp.107.114546 18359846 PMC2330294

[B10] Garciadeblás B. Senn M. E. Bañuelos M. A. Rodríguez-Navarro A. (2003). Sodium transport and HKT transporters: the rice model. *Plant J.* 34 788–801. 10.1046/j.1365-313X.2003.01764.x 12795699

[B11] Giehl R. F. Gruber B. D. von Wirén N. (2014). It’s time to make changes: modulation of root system architecture by nutrient signals. *J. Exp. Bot.* 65 769–778. 10.1093/jxb/ert421 24353245

[B12] Giehl R. F. von Wirén N. (2014). Focus issue on roots: root nutrient foraging. *Plant Physiol.* 166 509–517. 10.1104/pp.114.245225 25082891 PMC4213083

[B13] Gierth M. Maser P. Schroeder J. I. (2005). The potassium transporter AtHAK5 functions in K+ deprivation-induced high-affinity K+ uptake and AKT1 K+ channel contribution to K+ uptake kinetics in Arabidopsis roots. *Plant Physiol.* 137 1105–1114. 10.1104/pp.104.057216 15734909 PMC1065410

[B14] Gruber B. D. Giehl R. F. H. Friedel S. von Wirén N. (2013). Plasticity of the Arabidopsis root system under nutrient deficiencies. *Plant Physiol.* 163 161–179. 10.1104/pp.113.218453 23852440 PMC3762638

[B15] Guan P. Wang R. Nacry P. Breton G. Kay S. A. Pruneda-Paz J. L. (2014). Nitrate foraging by Arabidopsis roots is mediated by the transcription factor TCP20 through the systemic signaling pathway. *Proc. Natl. Acad. Sci. U.S.A.* 111 15267–15272. 10.1073/pnas.1411375111 25288754 PMC4210337

[B16] Gupta S. Yadav B. S. Raj U. Freilich S. Varadwaj P. K. (2017). Transcriptomic analysis of soil grown *T. aestivum* cv. root to reveal the changes in expression of genes in response to multiple nutrients deficiency. *Front. Plant Sci.* 8:1025. 10.3389/fpls.2017.01025 28690617 PMC5479913

[B17] Hafsi C. Debez A. Abdelly C. (2014). Potassium deficiency in plants: effects and signaling cascades. *Acta Physiol. Plant.* 36 1055–1070. 10.1007/s00709-015-0845-y 26085375

[B18] Haling R. E. Brown L. K. Stefanski A. Kidd D. R. Ryan M. H. Sandral G. A. (2018). Differences in nutrient foraging among *Trifolium subterraneum* cultivars deliver improved P-acquisition efficiency. *Plant Soil* 424 539–554. 10.1007/s11104-017-3511-7

[B19] Hassan Z. U. Arshad M. (2010). Cotton growth under potassium deficiency stress is influenced by photosynthetic apparatus and root system. *Pak. J. Bot.* 42 917–925.

[B20] Held K. Pascaud F. Eckert C. Gajdanowicz P. Hashimoto K. Corratgéfaillie C. (2011). Calcium dependent modulation and plasma membrane targeting of the AKT2 potassium channel by the CBL4/CIPK6 calcium sensor/protein kinase complex. *Cell Res.* 21 1116–1130. 10.1038/cr.2011.50 21445098 PMC3193494

[B21] Ho C. H. Tasy Y. F. (2010). Nitrate, ammonium and potassium sensing and signaling. *Curr. Opin. Plant Biol.* 13 604–610. 10.1016/j.pbi.2010.08.005 20833581

[B22] Hodge A. (2004). The plastic plant: root responses to heterogeneous supplies of nutrients. *New Phytol.* 162 9–24. 10.1111/j.1469-8137.2004.01015.x

[B23] Hong J. P. Takeshi Y. Kondou Y. Schachtman D. P. Matsui M. Shin R. (2013). Identification and characterization of transcription factors regulating Arabidopsis HAK5. *Plant Cell Physiol.* 54 1478–1490. 10.1093/pcp/pct094 23825216

[B24] Hong-Hermesdorfa A. Brüxa A. Grüberb A. Grüberb G. Schumacher K. (2006). A WNK kinase binds and phosphorylates V-ATPase subunit C. *FEBS Lett.* 580 932–939. 10.1016/j.febslet.2006.01.018 16427632

[B25] Jackson R. B. Caldwell M. M. (1993). The scale of nutrient heterogenity around individual plants and its quantification with geostatistics. *Ecology* 74 612–614. 10.2307/1939320

[B26] Jung J. Y. Ryoung S. Daniel P. S. (2009). Ethylene mediates response and tolerance to potassium deprivation in Arabidopsis. *Plant Cell* 21 607–621. 10.1105/tpc.108.063099 19190240 PMC2660615

[B27] Kassam A. Friedrich T. Derpsch R. (2018). Global spread of conservation agriculture. *Int. J. Environ. Stud.* 8:1494927 10.1080/00207233.2018.1494927

[B28] Kellermeier F. Chardon F. Amtmann A. (2013). Natural variation of Arabidopsis root architecture reveals complementing adaptive strategies to potassium starvation. *Plant Physiol.* 161 1421–1432. 10.1104/pp.112.211144 23329148 PMC3585606

[B29] Kim M. J. Ciani S. Schachtman D. P. (2010). A peroxidase contributes to ROS production during Arabidopsis root response to potassium deficiency. *Mol. Plant* 3 420–427. 10.1093/mp/ssp121 20139158

[B30] Kim M. J. Ruzika D. Shin R. Schachtman D. P. (2012). The Arabidopsis AP2/ERF transcription factor RAP2.11 modulates plant response to low-potassium conditions. *Mol. Plant* 5 1042–1057. 10.1093/mp/sss003 22406475

[B31] Krouk G. Mirowski P. Lecun Y. Shasha D. E. Coruzzi G. M. (2010). Predictive network modeling of the high-resolution dynamic plant transcriptome in response to nitrate. *Genome Biol.* 11:R123. 10.1186/gb-2010-11-12-r123 21182762 PMC3046483

[B32] Lee S. Sergeeva L. I. Vreugdenhil D. (2017). Natural variation of hormone levels in Arabidopsis roots and correlations with complex root architecture. *J. Integr. Plant Biol.* 60 292–309. 10.1111/jipb.12617 29205819 PMC5947113

[B33] Lee S. C. Lan W. Z. Kim B. G. Li L. Yong H. C. Pandey G. K. (2007). A protein phosphorylation/dephosphorylation network regulates a plant potassium channel. *Proc. Natl. Acad. Sci. U.S.A.* 104 15959–15964. 10.1073/pnas.0707912104 17898163 PMC2000415

[B34] Leigh R. A. Wyn Jones R. G. (1984). A hypothesis relating critical potassium concentrations for growth to the distribution and function of this ion in the plant cell. *New Phytol.* 97 1–13. 10.1111/j.1469-8137.1984.tb04103.x

[B35] Li D. Nan H. Liang J. Cheng X. Zhao C. Z. Yin H. J. (2017). Responses of nutrient capture and fine root morphology of subalpine coniferous tree *Picea asperatato* nutrient heterogeneity and competition. *PLoS One* 12:e0187496. 10.1371/journal.pone.0187496 29095947 PMC5667764

[B36] Liu J. Han L. Chen F. Bao J. Zhang F. Mi G. (2008). Microarray analysis reveals early responsive genes possibly involved in localized nitrate stimulation of lateral root development in maize (*Zea mays* L.). *Plant Sci.* 175 272–282. 10.1016/j.plantsci.2008.04.009

[B37] Liu Y. Donner E. Lombi E. Li R. Wu Z. Zhao F. J. (2013). Assessing the contributions of lateral roots to element uptake in rice; using an auxin-related lateral root mutant. *Plant Soil* 372 125–136. 10.1007/s11104-012-1582-z

[B38] Ma Q. Rengel Z. Bowden B. (2007). Heterogeneous distribution of phosphorus and potassium in soil influences wheat growth and nutrient uptake. *Plant Soil* 291 301–309. 10.1007/s11104-007-9197-5

[B39] Ma T. L. Wu W. H. Wang Y. (2012). Transcriptome analysis of rice root responses to potassium deficiency. *BMC Plant Biol.* 12:161. 10.1186/1471-2229-12-161 22963580 PMC3489729

[B40] Mcnickle G. G. St Clair C. C. Cahill J. F. (2009). Focusing the metaphor: plant root foraging behaviour. *Trends Ecol. Evol.* 24 419–426. 10.1016/j.tree.2009.03.004 19409652

[B41] Mills H. A. Jones J. B. (1996). *Plant Analysis Handbook*, 2nd Edn Athens: Micro-Macro Press, 45–48.

[B42] Mittler R. Vanderauwera S. Gollery M. Van B. F. (2004). Reactive oxygen gene network of plants. *Trends Plant Sci.* 9 490–498. 10.1016/j.tplants.2004.08.009 15465684

[B43] Mugnai S. Marras A. M. Mancuso S. (2011). Effect of hypoxic acclimation on anoxia tolerance in Vitis roots: response of metabolic activity and K+ fluxes. *Plant Cell Physiol.* 52 1107–1116. 10.1093/pcp/pcr061 21551160

[B44] Paterson E. Sim A. Standing D. Dorward M. Mcdonald A. J. (2006). Root exudation from *Hordeum vulgare* in response to localized nitrate supply. *J. Exp. Bot.* 57 2413–2420. 10.1093/jxb/erj214 16766600

[B45] Pottosin I. (2014). Regulation of potassium transport in plants under hostile conditions: implications for abiotic and biotic stress tolerance. *Physiol. Plant.* 151 257–279. 10.1111/ppl.12165 24506225

[B46] Revel A. T. Talaat A. M. Norgard M. V. (2002). DNA microarray analysis of differential gene expression in *Borrelia burgdorferi*, the Lyme disease spirochete. *Proc. Natl. Acad. Sci. U.S.A.* 99 1562–1567. 10.1073/pnas.032667699 11830671 PMC122230

[B47] Richard M. Bernhardt T. Bell G. (2000). Environmental heterogeneity and the spatial structire of fern species diversity in one hectare of old-growth forest. *Ecography* 23 231–245. 10.1111/j.1600-0587.2000.tb00279.x

[B48] Robaglia C. Thomas M. Meyer C. (2012). Sensing nutrient and energy status by SNRK1 and TOR kinases. *Curr. Opin. Plant Biol.* 15 301–307. 10.1016/j.pbi.2012.01.012 22305521

[B49] Römheld V. Kirkby E. A. (2010). Research on potassium in agriculture: needs and prospects. *Plant Soil* 335 155–180. 10.1007/s11104-010-0520-1

[B50] Ruan L. Wei K. Wang L. Cheng H. Zhang F. Wu L. (2016). Characteristics of NH4+ and NO3- fluxes in tea (*Camellia sinensis*) roots measured by scanning ion-selective electrode technique. *Sci. Rep.* 6:38370. 10.1038/srep38370 27918495 PMC5137579

[B51] Ruan L. Zhang J. B. Xin X. L. Zhang C. Z. Ma D. H. Chen L. (2015). Comparative analysis of potassium deficiency-responsive transcriptomes in low potassium susceptible and tolerant wheat (*Triticum aestivum* L.). *Sci. Rep.* 5:10090. 10.1038/srep10090 25985414 PMC4650753

[B52] Ruffel S. Coruzzi G. M. Ristova D. Shasha D. Birnbaum K. D. Coruzzi G. M. (2011). Nitrogen economics of root foraging: transitive closure of the nitrate-cytokinin relay and distinct systemic signaling for n supply vs. demand. *Proc. Natl. Acad. Sci. U.S.A.* 108 18524–18529. 10.1073/pnas.1108684108 22025711 PMC3215050

[B53] Schachtman D. P. Shin R. (2007). Nutrient sensing and signaling: NPKS. *Annu. Rev. Plant Biol.* 58 47–69. 10.1146/annurev.arplant.58.032806.103750 17067284

[B54] Shabnam R. Iqbal M. T. (2016). Understanding phosphorus dynamics on wheat plant under split-root system in alkaline soil. *Braz. J. Sci. Technol.* 3:19 10.1186/s40552-016-0031-6

[B55] Shen J. Li H. Neumann G. Zhang F. (2005). Nutrient uptake, cluster root formation and exudation of protons and citrate in *Lupinus albus*, as affected by localized supply of phosphorus in a split-root system. *Plant Sci.* 168 837–845. 10.1016/j.plantsci.2004.10.017

[B56] Shin R. Berg R. H. Schachtman D. P. (2005). Reactive oxygen species and root hairs in Arabidopsis root response to nitrogen, phosphorus and potassium deficiency. *Plant Cell Physiol.* 46 1350–1357. 10.1093/pcp/pci145 15946982

[B57] Shin R. Schachtman D. P. (2004). Hydrogen peroxide mediates plant root cell response to nutrient deprivation. *Proc. Natl. Acad. Sci. U.S.A.* 101 8827–8832. 10.1073/pnas.0401707101 15173595 PMC423280

[B58] Singh P. Blanke M. M. (2000). Deficiency of potassium but not phosphorus enhances root respiration. *Plant Growth Regul.* 32 77–81. 10.1023/A:1006397611793

[B59] Stoimenova M. Igamberdiev A. U. Gupta K. J. Hill R. D. (2007). Nitrite-driven anaerobic ATP synthesis in barley and rice root mitochondria. *Planta* 226 465–474. 10.1007/s00425-007-0496-0 17333252

[B60] Sun B. Gao Y. Lynch J. (2018). Large crown root number improves topsoil foraging and phosphorus acquisition. *Plant Physiol.* 177 90–104. 10.1104/pp.18.00234 29618638 PMC5933112

[B61] Szczerba M. W. Britto D. T. Kronzucker H. J. (2009). K+ transport in plants: physiology and molecular biology. *J. Plant Physiol.* 166 447–466. 10.1016/j.jplph.2008.12.009 19217185

[B62] Tabata R. Sumida K. Yoshii T. Ohyama K. Shinohara H. Matsubayashi Y. (2014). Perception of root-derived peptides by shoot LRR-RKs mediates systemic N-demand signaling. *Science* 346 343–346. 10.1126/science.1257800 25324386

[B63] Trevisan S. Manoli A. Ravazzolo L. Botton A. Pivato M. Masi A. (2015). Nitrate sensing by the maize root apex transition zone: a merged transcriptomic and proteomic survey. *J. Exp. Bot* 66 3699–3715. 10.1093/jxb/erv165 25911739 PMC4473975

[B64] Wang Y. Wu W. H. (2013). Potassium transport and signaling in higher plants. *Annu. Rev. Plant Biol.* 64 451–476. 10.1146/annurev-arplant-050312-120153 23330792

[B65] Williams A. Davis A. S. Jilling A. Grandy A. S. Koide R. T. Mortensen D. A. (2017). Reconciling opposing soil processes in row-crop agroecosystems via soil functional zone management. *Agric. Ecosyst. Environ.* 236 99–107. 10.1016/j.agee.2016.11.012

[B66] Wu L. H. Wang Y. Nie J. Fan X. H. Cheng Y. Y. (2013). A network pharmacology approach to evaluating the efficacy of Chinese medicine using genome-wide transcriptional expression data. *Evid. Based Complement. Alternat. Med.* 2013:915343. 10.1155/2013/915343 23737854 PMC3666440

[B67] Zeng Q. Zhang P. Wu Z. Xue P. Lu D. Ye Z. (2014). Quantitative proteomics reveals ER-α involvement in CD146-induced epithelial-mesenchymal transition in breast cancer cells. *J. Proteomics* 103 153–169. 10.1016/j.jprot.2014.03.033 24704855

[B68] Zhang J. Yu H. Zhang Y. Wang Y. Li M. Zhang J. (2016). Increased abscisic acid levels in transgenic maize overexpressing AtLOS5 mediated root ion fluxes and leaf water status under salt stress. *J. Exp. Bot.* 67 1339–1355. 10.1093/jxb/erv528 26743432 PMC4762378

[B69] Zhao X. Liu S. L. Pu C. Zhang X. Q. Xue J. F. Ren Y. X. (2017). Crop yields under no-till farming in china: a meta-analysis. *Eur. J. Agric.* 84 67–75. 10.1038/nature13809 25337882

[B70] Zhao X. H. Hai-Qiu Y. U. Wen J. Wang X. G. Qi D. U. Wang J. (2016). Response of root morphology, physiology and endogenous hormones in maize (*Zea mays* L.) to potassium deficiency. *J. Integr. Agric.* 15 785–794. 10.1016/S2095-3119(15)61246-1

[B71] Zheng C. Huang Y. C. Liang J. H. Feng Z. Zhu Y. G. (2012). A novel sediment microbial fuel cell with a biocathode in the rice rhizosphere. *Bioresour. Technol.* 108 55–59. 10.1016/j.biortech.2011.10.040 22265978

[B72] Zhou C. Jiang W. Li Y. Hon X. Liu A. Cai L. (2017). Morphological plasticity and phosphorus uptake mechanisms of hybrid eucalyptus roots under spatially heterogeneous phosphorus stress. *J. For. Res.* 28 713–724. 10.1007/s11676-016-0335-x

[B73] Zhu Z. Li C. Zeng Y. Ding J. Qu Z. Gu J. (2016). PHB associates with the HIRA complex to control an epigenetic-metabolic circuit in human ESCs. *Cell Stem Cell* 20 274–289. 10.1016/j.stem.2016.11.002 27939217

