# Supplementary material for: Potential Root Foraging Strategy of Wheat (Triticum aestivum L.) for Potassium Heterogeneity
Source: Front Plant Sci. 2018 Nov 27;9:1755. doi: 10.3389/fpls.2018.01755 (PMC6277704; doi:10.3389/fpls.2018.01755)
Supplement: TABLE S1 — Primers used in qRT-PCR experiments. [file Table_1.docx]

**Supplementary Table S1**: Primers used in qRT-PCR experiments.

| **Probe_Set_ID** | **Forward primer (5'-3')** | **Reverse primer (5'-3')** |
| --- | --- | --- |
| Ta.25219.1.A1_at | GTGCCGTGGGAGGATTTCG | GCAGTGCCGTTCTGTTTGTTTC |
| Ta.113.1.S1_at | TGCCAGGACAAACCATACAGC | GCCTTCCATAGAGCATGACCA |
| Ta.92.1.S1_s_at | ATGACCCTGACCGGCGTGTA | GTCAACTGCCAATGGAACTGC |
| Ta.593.2.S1_x_at | GGGAGCGTGCTACTTCATCTAC | CAAGACCAGCAACAACGAAATG |
| Ta.6556.1.S1_x_at | AAGAATCCTACAAGGACAGCACC | GGAAGTCCAGAGGGTCAAGTTATC |
| Ta.30726.1.S1_at | TGTTCGGTTGCTTGATGTAGTAG | CACGCATTATAAATTTTGGG |
| Ta.30668.1.S1_at | CAGCATGATCCCTATTTGTGAG | TAACACGCTTGTTAACGCTTG |
| Ta.22338.2.S1_a_at | AGAAGAGAACGTCTGTCATCTGC | ACGTTCTCCTCTTACTCAGAGTCT |
| Ta.9143.1.S1_at | CTACGATTTCCATGTTCCAGTG | CCGTTCTGGACGTAGCAAAAC |
| Ta.1207.1.S1_s_at | ATCTTGTTGCTTACGGGAGGA | GTGTAGAAAGTGTTGCGGTCAT |
| Ta.3305.2.S1_a_at | CACCGGATGCCAAGATGTTC | CCTTCAGCTTGACGACGGACT |
| Ta.4969.1.S1_x_at | GACCACCTCAAGAACACCTCC | GCAGCGAAGCGCAATACTAC |
