# Supplementary material for: Potential Root Foraging Strategy of Wheat (Triticum aestivum L.) for Potassium Heterogeneity
Source: Front Plant Sci. 2018 Nov 27;9:1755. doi: 10.3389/fpls.2018.01755 (PMC6277704; doi:10.3389/fpls.2018.01755)
Supplement: TABLE S2 — Root morphological characteristics under potassium (K) heterogeneity. [file Table_2.docx]

**Supplementary Table S2:** Root morphological characteristics under potassium (K) heterogeneity.

| **Treatments** | **Total root length (cm)** | **Total root surface area (cm^2^)** | **Root tips** | **Total root length / Root tips (cm)** |
| --- | --- | --- | --- | --- |
| Sp_0_. NK | 802.33 ± 19.71b | 97.01 ± 1.07b | 693 ± 7.97b | 1.16 ± 0.04b |
| Sp_0_. MK | 768.94 ± 6.3b | 87.51 ± 1.43c | 642 ± 8.37c | 1.2 ± 0.03b |
| Sp. NK | 1086.24 ± 45.94a | 111.12 ± 3.37a | 763 ± 13.17a | 1.42 ± 0.06a |
| Sp. LK | 624.12 ± 12.93c | 62.96 ± 0.87d | 557 ± 9.62d | 1.12 ± 0.04b |

Note: This table showed two types of K heterogeneity as follows: (1) Sp_0_. MK/Sp_0_. NK: one compartment had 0.5 mmol L^-1^ K_2_SO_4_, and the other had 1.0 mmol L^-1^ K_2_SO_4_; (2) Sp. LK/Sp. NK: one compartment had 0.005 mmol L^-1^ K_2_SO_4_, and the other had 1.0 mmol L^-1^ K_2_SO_4_. Data were means ± SE (n = 3). Different letters showed significant differences at the level of *P* < 0.05.
