# Supplementary material for: Potential Root Foraging Strategy of Wheat (Triticum aestivum L.) for Potassium Heterogeneity
Source: Front Plant Sci. 2018 Nov 27;9:1755. doi: 10.3389/fpls.2018.01755 (PMC6277704; doi:10.3389/fpls.2018.01755)
Supplement: TABLE S3 — Potential key genes involved in the regulation of wheat root responses to K heterogeneity. [file Table_3.docx]

**Supplementary Table S3:** Potential key genes involved in the regulation of wheat root responses to K heterogeneity.

| **Up-regulated genes in Sp. NK vs C. NK** | | **Down-regulated genes in Sp. LK vs C. LK** | |
| --- | --- | --- | --- |
| Iron ion binding | Ta.1457.1.S1_x_at | Iron ion binding | Ta.21061.3.S1_at |
|  | Ta.3529.1.S1_at |  | Ta.5546.3.S1_a_at |
|  | Ta.18497.1.S1_at |  |  |
|  | Ta.6801.1.A1_at |  |  |
| Mitochondrion | Ta.3529.1.S1_at | Mitochondrion | Ta.27139.1.S1_x_at |
|  | Ta.4593.1.A1_at |  | TaAffx.88356.1.S1_at |
|  | TaAffx.9460.1.S1_at |  | Ta.943.1.A1_x_at |
|  | Ta.10549.1.A1_x_at |  | Ta.24720.2.S1_at |
|  | Ta.10549.1.A1_at |  | Ta.23081.1.S1_x_at |
| Transcription factor activity | TaAffx.9231.2.S1_s_at | Transcription factor activity | TaAffx.70539.1.A1_at |
|  | TaAffx.9231.2.S1_x_at |  | Ta.8614.1.S1_at |
|  | Ta.4678.3.S1_x_at |  | TaAffx.128540.1.S1_at |
|  | Ta.9239.1.A1_x_at |  | Ta.27144.1.S1_a_at |
|  | Ta.16082.2.S1_x_at |  |  |
|  | Ta.17378.1.S1_at |  |  |
|  | Ta.16082.1.A1_at |  |  |
| Cytoplasmic membrane-bounded vesicle | Ta.303.2.S1_x_at | Cytoplasmic membrane-bounded vesicle | TaAffx.128795.28.S1_at |
|  | Ta.23127.2.S1_x_at |  | Ta.3232.1.S1_a_at |
|  | TaAffx.130164.1.S1_x_at |  | Ta.25583.1.S1_at |
|  | Ta.1763.1.S1_x_at |  | Ta.7883.1.S1_x_at |
|  | Ta.29367.1.S1_at |  | Ta.4058.1.S1_at |
|  | Ta.8600.1.S1_s_at |  | TaAffx.56333.1.S1_at |
|  | Ta.13988.1.S1_at |  | Ta.9697.1.S1_s_at |
|  | Ta.23127.1.S1_at |  | Ta.1290.1.S1_x_at |
|  | Ta.19463.1.S1_at |  | Ta.13938.1.S1_at |
|  | TaAffx.98425.1.S1_s_at |  | TaAffx.560.3.S1_at |
|  |  |  | Ta.1290.1.S1_a_at |
| Plastid | TaAffx.24253.1.S1_s_at | Plastid | Ta.28907.2.S1_x_at |
|  | Ta.103.1.S1_at |  | TaAffx.124056.1.S1_at |
|  | TaAffx.117450.1.S1_at |  | Ta.5546.1.S1_a_at |
|  | TaAffx.98004.1.S1_at |  | Ta.113.1.S1_at |
|  | Ta.25405.2.S1_a_at |  | Ta.1995.1.S1_at |
| Calcium ion binding | Ta.2882.1.S1_at |  | Ta.8531.1.A1_at |
|  | Ta.23032.2.S1_x_at |  | Ta.23081.2.S1_a_at |
|  | Ta.11016.1.S1_at |  | Ta.10832.1.S1_at |
| Glutathione transferase | Ta.3681.1.S1_x_at |  | Ta.25578.1.S1_a_at |
|  | Ta.3679.1.S1_x_at |  | Ta.1995.3.S1_a_at |
|  | Ta.1457.1.S1_x_at | Methyltransferase | Ta.5529.1.A1_x_at |
|  | Ta.303.2.S1_x_at | Protein amino acid phosphorylation | Ta.27859.1.A1_at |
| Cellular respiration | Ta.22678.1.A1_s_at |  | TaAffx.102420.1.S1_at |
| Jasmonic acid mediated signaling pathway | Ta.25405.2.S1_a_at | Potassium ion transport | Ta.113.1.S1_at |
| Respiratory gaseous exchange | Ta.28112.1.S1_at |  | TaAffx.52479.1.S1_at |
|  |  | Protein serine/threonine kinase activity | Ta.27576.1.A1_at |
|  |  |  | Ta.5331.1.A1_x_at |
|  |  | Oxygen transport | Ta.14486.1.S1_at |
